# Extracellular vesicles: the double-edged sword in viral infections

**DOI:** 10.1128/mbio.03316-25

**Published:** 2025-12-19

**Authors:** Sharda Kumari, Arup Banerjee

**Affiliations:** 1 Laboratory of Virology, Regional Centre for Biotechnology, NCR Biotech Science 682813 , Faridabad, India; Albert Einstein College of Medicine, Bronx, New York, USA

**Keywords:** extracellular vesicles, EVs, viral infection, nanocarriers, HIV, SARS-CoV-2, dengue, pathogenesis, immune modulation, vaccines

## Abstract

Extracellular vesicles (EVs) are lipid-bound nanocarriers released by various eukaryotic cells and found in diverse bodily fluids. EVs have transitioned from being considered cellular waste disposers to significant players in intercellular communication and signaling. These EVs carry signature cargos of infected cells and thus can be helpful as biomarkers or prognostic markers for infectious diseases. Viruses can manipulate the EV biogenesis machinery in their own dissemination. EVs released from virus-infected cells can carry immune modulatory molecules, thus contributing to disease progression. This comprehensive review collates the information on the impact of EVs on viral infection and disease progression.

## INTRODUCTION

Extracellular vesicles (EVs) are small membrane vesicles released from almost all types of eukaryotic cells and are detectable both in human and animal body fluids. EVs were discovered by Pan and Johnstone (1) in the process of platelet differentiation (1). Earlier, it was thought to be like garbage bags to remove unnecessary materials from the cell, but further studies and characterization of the contents of these nanovesicles showed the presence of MHC molecules, which indicated their role in antigen presentation (2–4). EVs are classified as apoptotic bodies (500–2,000 nm), microvesicles (100–1,000 nm), and exosomes (30–150 nm); they differ in size, content, and their origination process. Apoptotic cells produce apoptotic bodies by cell blebbing, whereas EVs are derived from healthy cells, which play a vital role in maintaining homeostasis of the cells. Microvesicles are the result of outward budding of the plasma membrane. Another class of EVs, exosomes, originates from the endosomal system, a complex network comprising distinct compartments, including early endosomes, late endosomes, and recycling endosomes. These endosomes are formed through the invagination of the plasma membrane. The early endosomes transform into intraluminal vesicles (ILVs) and fuse to form MVBs (multivesicular bodies); during this process, cytosolic proteins, nucleic acids, and lipids are sorted into these small vesicles with the help of multi-subunit protein machinery known as endosomal sorting complexes required for transport (ESCRT) (5). It can be produced in an ESCRT-dependent and an ESCRT-independent manner (6, 7). However, it is not clear what conditions overpower biogenesis pathways over one another. MVBs encompass ILVs spanning a size spectrum of 30–100 nm.

EVs may be released from “donor” cells through multiple processes like endocytosis, lipid raft-mediated endocytosis, membrane fusion, and caveolae-mediated endocytosis (8–10).

Tetraspanins like CD63 and CD81 are also involved in biosynthesis and sorting specific proteins into EVs (11). Interestingly, viruses use similar pathways for their assembly and egress. This leads to the possibility of being packaged as a viral genome or protein within the vesicles. These EVs are taken up by the recipient cell by phagocytosis, micropinocytosis, endocytosis, and the fusion process (12) and often play a crucial role in promoting viral transmission (13), replication (14), and enhancing the virus’s ability within the hostile environment of the host immune response (15) and dictate the outcome of the infection. In addition to facilitating viral infection, EVs can provoke an immune response against viruses (16).

EVs have grabbed attention in the past few decades due to their cargo, which can be different nucleic acids, proteins, lipids, and carbohydrates (17–22). The composition of EVs varies depending on their origin and how they form within cells. EVs are produced constitutively and can be retrieved from circulating body fluids, namely saliva, milk, plasma, serum, and cerebrospinal fluid (CSF) (17, 23); therefore, they are considered an essential source of biomarkers for different diseases. In this review article, we would like to comprehensively review the recent understanding of the class of exosomes and microvesicles (EVs) in viral infection and shed light on the dual role of these carriers in disease progression.

## THE POTENTIAL FATE OF CROSSTALK BETWEEN THE VIRUS AND THE EVS BIOGENESIS PATHWAY

**Fig 1:**
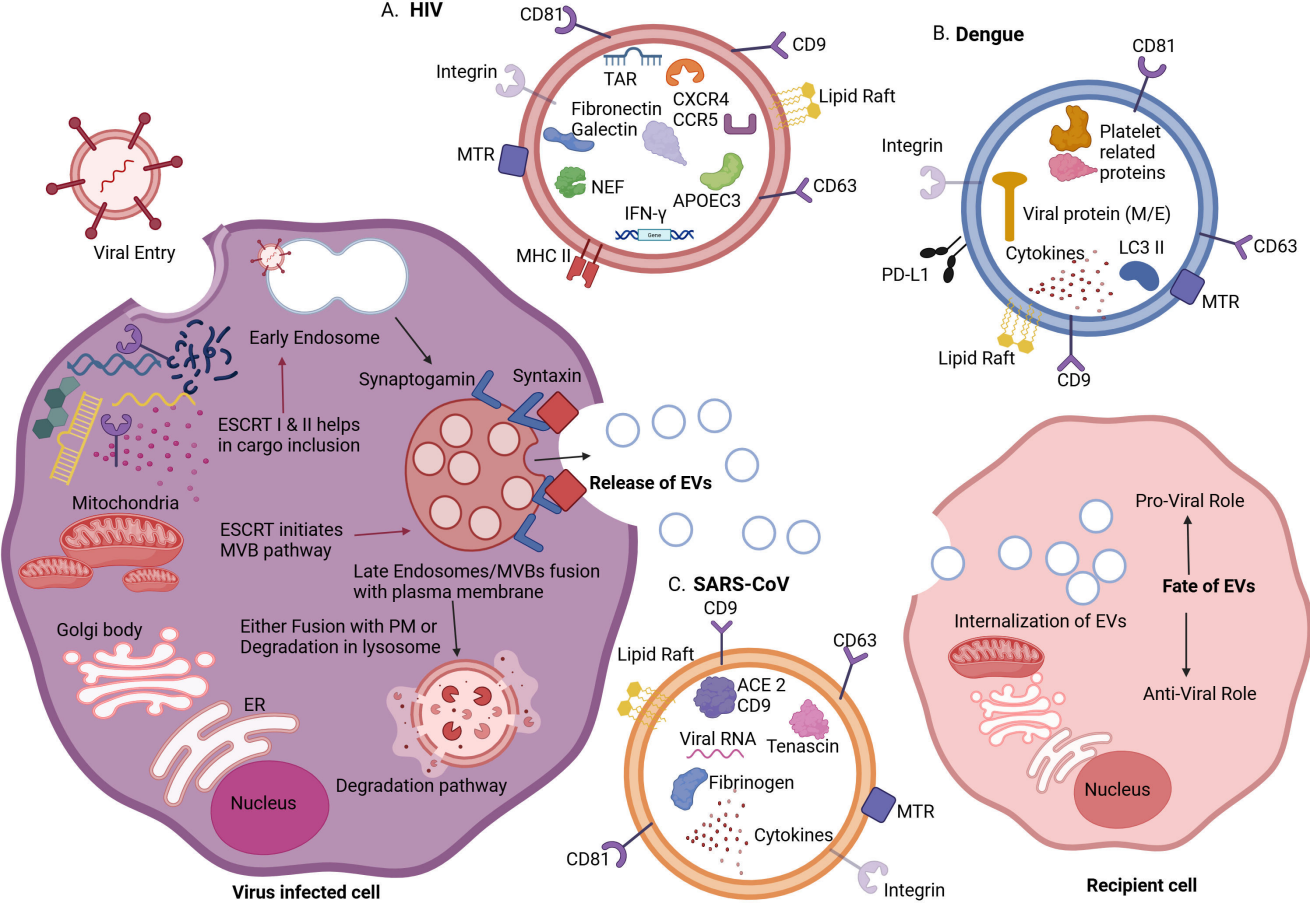
Schematic representation of the involvement of ESCRT machinery in the biogenesis of EVs in different viral infections. Viruses manipulate the EV biogenesis and cargo loading. Here, three examples are shown: HIV, dengue virus, and SARS-CoV-2. After loading manipulated cargo from virus-infected cells, these EVs can show the pro-viral or antiviral role on the recipient cells’ fate.

As several viruses enter via the endocytic pathway, and one class of EV “exosomes” originates in the endocytic compartment, the virus can command this pathway in its own favor. The virus enters the cell by different methods, such as endocytosis and fusion with the membrane. After the virus is assembled in infected cells, it can egress from the cell membrane by budding (24). Enveloped virions are especially likely to hijack multiple components of ESCRT machinery (TSG101 of ESCRT-I, ALIX, and NEDD4L), which aid in dividing the plasma membrane to facilitate internalization and packaging (Fig. 1). Different viruses are reported to interact with ESCRT components and use them in their replication and spread (25–27).

The interplay between viruses and the pathways involved in EV biogenesis can influence various aspects of virus release and the immune response. Moreover, this interaction might speed up viral replication (28). The mechanisms involved in EVs' biogenesis likely offer a conducive environment that supports viral infection and propagation. Also, the EV particles have been found to contain viral components from various other types of viruses, such as HCV (14), JEV (29), HIV (30), EBV (31), HSV-1 (32), and Kaposi sarcoma herpes virus (32, 33).

What component of the viruses facilitated interaction with ESCRT machinery? Numerous investigations have shown that viral structural proteins incorporate distinct signature motifs (such as P(T/S)AP, YPXL, and PPXY), enabling them to engage with the ESCRT complex (34). For instance, the P(T/S)AP motif is identified within the viral gag protein of retroviridae, like HIV and murine leukemia virus, Z proteins found in Arenaviridae, VP40 proteins present in Filoviridae, along with Matrix proteins seen in Rhabdoviridae (35–39) activate TSG101, leading to the participation of the ESCRT complex at the site of virus entry in an ESCRT-dependent way (24). Interestingly, host cellular proteins also exploit these ESCRT-interacting motifs during exosome biogenesis, indicating that both viral and exosomal release share overlapping ESCRT-dependent mechanisms.

Many viruses belong to different families, like Hepadnaviridae, Flaviviridae, and Tombusviridae, which are potent to activate the MVB pathway for viral particle entry in an ESCRT-independent process (39–42). The interaction of the viral YP_n_XL, PPXY motif (Filoviridae and Paramyxoviridae) with the V-domain of Alix, NEDD4L subunit, and E3 ubiquitin ligases can be exploited in favor of sorting of viral proteins into the MVBs (42, 43). Similarly, the association between PPXY and NEDD4 enhances the process of viral budding into MVBs (24, 35, 38, 39, 42).

Viruses such as respiratory syncytial, Hantavirus, and Influenza viruses have been shown to leverage the Rab pathway to facilitate their transport through the plasma membrane during viral egress. These viruses hijack the Rab GTPase complexes and associated effectors to promote the budding and release of newly formed viral particles from infected cells (44–47). In the case of EBV, it is shown that the LMP1 protein of the virus directly interacts with CD63, promoting cargo sorting and triggering the incorporation of viral particles in exosomes (48).

Exosomes also carry specific interferon-stimulated genes (ISGs), enhancing antiviral defense responses. For example, hepatocyte-derived exosomes can transfer the cytosolic RNA helicase DDX60 to natural killer (NK) cells, which facilitates the breakdown of HBV RNA. Similarly, Tetherin (BST2), an ISG known to restrict the release of HCV virions, can be packaged within exosomes and delivered to recipient cells to exert its antiviral function (49). Recent studies on human T-cell leukemia virus (HTLV) have provided preliminary insights into how this retrovirus modulates EV properties. Using ultracentrifugation-based isolation**,** researchers have reported subtle variations in EV size distribution and molecular composition derived from HTLV-infected cells compared to uninfected counterparts. These EVs often contain viral proteins such as Tax and p19 and altered host factors involved in immune regulation and cellular signaling. Although the available data remain limited, these findings suggest that HTLV infection may influence EV biogenesis and cargo sorting**,** contributing to viral persistence and intercellular communication (50–52).

Also, a study demonstrated that exosomes released from CD4^+^ T cells express surface CD4 molecules capable of binding to the HIV-1 envelope glycoprotein, thereby interfering with viral engagement of uninfected target cells. Furthermore, it was shown that the presence of the HIV-1 accessory protein Nef diminishes CD4 incorporation into exosomes, consequently reducing their decoy potential and enhancing viral infectivity (53).

In the context of neurotropic viruses, for example, Japanese encephalitis virus (JEV), EVs play a vital role in virus propagation by facilitating viral entry and assembly‐release. EVs isolated from JEV-infected permissive cells contain the virions, genome, and proteins of JEV, which could infect host cells. Also, virion-encapsulated EVs enhanced viral capability to cross the blood-brain barrier (29).

In general, viruses being smart can manipulate the EVs biogenesis process and utilize it to disseminate their own virulence to the host’s naïve cell. Therefore, it is crucial to understand this crosstalk to develop methods for disrupting the viral propagation.

## UNTANGLING THE DANCE: THE CLEVER USE OF EVS BY THE MANIPULATOR VIRUS

Owing to the similarities in the processes of EV biogenesis and virus replication, coupled with shared pathways, it is hypothesized that viruses could co-opt the mechanisms responsible for the EVs’ formation. This would enable them to propagate and evade immune cells more effectively. EVs can carry complete virions (either as singular units or aggregates), separate viral components, and even genetic material without its protective covering. Coating viruses with a lipid layer from the host cell facilitates their entry into other cells through exosomal ligands, enabling them to evade immune cells’ direct and indirect action.

The encapsulation of rotavirus and norovirus within EVs marked by CD9, CD81, and CD63 has been demonstrated, potentially elevating the likelihood of infection (54, 55).

Conversely, the internalization of EVs-loaded viral particles by antigen-presenting cells can enhance the antigen presentation process. Consequently, the EV-mediated transportation of specific viruses via EVs can be a double-edged sword within the intricate landscape of viral infections. Different viruses use EV to their advantage, as given in Table 1. Overall, EVs can play a complex and context-dependent role in viral infections, as facilitators or inhibitors of viral replication and dissemination. One report suggested that EVs derived from virus-infected cells, patients’ plasma, CSF, and serum can change the microenvironment by inducing proliferation, apoptosis, and immune escape of the virus (56). But the mechanism is ill-understood. As the virus poses major serious health threats all over the globe, it smartly seizes the EVs biogenesis mechanism in its own service for its replication, evasion, and immune evasion. Thus, studying EVs can provide new avenues to enhance our understanding of viral infection and its pathogenesis. Further research is needed to comprehensively understand the mechanisms underlying EVs’ dual nature in different viral infections and their potential for therapeutic interventions.

**TABLE 1:** Viral messengers: implications of viral cargo on naïve cells

Virus	Cargo	Function	Reference(s)
CHIKV	RNA elements	Increasing infectivity	(57)
Tick-borne flavivirus	Viral RNA and protein	Infection spread	(58)
EBV	RNA, miRNA, LMP1, 2A, gp350, EBERs galectin-9 (host factor)	Viral reactivation, apoptosis in T cells	(59–61)
Enterovirus 71	Whole virus, viral protein, RNA	Productive infection in human neuroblastoma cells, partial resistance to AB-neutralization	(62, 63)
HEV	Viral RNA, miRNA, ceramide, sphingomyelinase	Viral entry	(64)
Rift Valley fever virus	Viral proteins, miRNA, mRNA	Apoptosis, enhance infectivity	(65)
HIV	Viral protein, miRNA, host factors	To make CD4^+^ more permissible, spread in viral infectivity and other HIV-related diseases	(66)
Hepatitis A virus	HAV virions	To increase viral infectivity	(67, 68)
HTLV-1	Viral protein (Tax), mRNA	Induces proinflammatory cytokine production, generates Th1 subset	(69–71)
Herpes virus	Virion encapsulated in MVB	–^*a*^	(72)
Japanese encephalitis virus	Let 7i in EV	Induces neuronal death upon uptake	(73)

^*a*^ “–,” no available data.

## EVs: UNDERSTANDING THE INTRIGUING CONNECTION TO VIRAL INFECTION

In this review, the term EVs has been used as a general descriptor encompassing exosomes, microvesicles, and other secreted vesicles, unless explicitly specified in the cited study. It is essential to acknowledge that many publications describing EV-mediated effects on viral infections have not strictly separated exosomes from microvesicles due to the overlapping biophysical characteristics and the inherent technical challenges associated with their isolation and characterization. Therefore, we have retained the terminology (“exosomes” or “EVs”) as used by the original authors to maintain consistency with the primary literature.

### Role of EVs in HIV infection

HIV belongs to the retroviridae, having positive single-stranded RNA. The main target of HIV is dendritic cells, monocytes, macrophages, and T lymphocytes bearing CD4. Their size is slightly larger than EVs, which removes the idea of mature HIV being packed in EVs. HIV and EVs share converging pathways for their formation and release (74–76). Also, for its assembly, HIV completely takes over Rab27a pathways (77, 78).

HIV can also transfer TAR RNA to the uninfected cells through the exosomal route, which interacts with the tat protein to produce HIV virions (79). The presence of Nef in EVs represses the antiviral response against HIV in uninfected cells (80, 81). Nef proteins (accessory protein of HIV) released from infected cells into exosomes can impair cholesterol metabolism in the recipient’s cells, which augments the inflammation and co-morbidities in HIV patients (82). Nef in exosomes is also reported to reduce the CD4^+^ EVs release from CD4^+^ T cells to reduce their recognition by the host immune system (66, 83). HIV-1 Nef facilitates viral replication and contributes to pathogenesis by inducing the depletion of CD4 and MHC-1 molecules. Nef achieves this by binding to the cytosolic tail of CD4 and MHC-1 proteins, subsequently interfering with their intracellular trafficking. Nef redirects these proteins toward MVBs and subsequently toward lysosomes, where they undergo degradation (84).

Also, these Nef-bearing EVs were shown to inhibit the production of IgG and IgA, thereby hampering the humoral immune response (85). The presence of Nef proteins in exosomes is also associated with the increased risk of neurological disorders like Alzheimer’s disease by activating inflammatory cytokines in microglial cells, as EVs can cross the blood-brain barrier (86, 87). These inflammatory cytokines are responsible for the generation of free radicals and for causing oxidative stress. EVs isolated from biological fluids like plasma, blood, and cells infected with HIV showed the presence of co-receptors of HIV, CCR5, and CXCR4 receptors, which can be transferred to naïve cells and render them susceptible to viral entry (88, 89). Although *in vitro* studies have reported the presence of HIV co-receptors CCR5 and CXCR4 in EVs and their transfer to naïve cells, thereby potentially increasing viral susceptibility, the *in vivo* significance of this mechanism remains to be fully established. HIV infection in physiological conditions continues to exhibit cell-type specificity, suggesting that EV-mediated receptor transfer may have limited functional impact *in vivo*. Exosomes derived from infected CD4^+^ T cells can reactivate the latent HIV in the macaque model (90). Exosomes derived from HIV-infected cells have also been shown to carry HIV miRNAs like vmiR99 and vmiR88, which increase TNF-α production in macrophages (91, 92).

EVs released from HIV-infected cells showed the presence of MHC-II, CD45, and CD86, which help reduce the host’s antiviral immune response and support HIV replication (93). Incubation of patient-derived exosomes with PBMC showed increased levels of CD38 on memory CD8^+^ T cells and CD4^+^ T cells, showing increased inflammation (94). Proteomics study of HIV-infected DC-derived exosomes showed the presence of fibronectin and galectin-3, which induce proinflammatory cytokines in the recipient cells, thereby establishing their role in pathogenesis (95).

One recent study has shown that HIV-1 RNA is encapsulated within EVs in both CSF and serum of individuals on antiretroviral therapy. Importantly, higher EV-associated HIV-1 RNA levels in the CSF correlate with neurocognitive impairment, suggesting that EVs contribute to persistent viral transcription and central nervous system dysfunction despite effective viral suppression (96).

Whereas opposing reports are also available that support the antiviral role of EVs released from infected cells. A report showed that infected CD8^+^ T cells derived exosomes inhibit the HIV transcription into the recipient cells (97). Interferon genes released in exosomes from TLR3-activated brain microvascular endothelial cells also showed an antiviral response in the recipient cells against HIV (98). The transfer of APOEC3 into the exosomes from the infected cells to uninfected cells suppressed HIV replication by catalyzing the conversion of cytosine residue to uracil within the minus strand of viral DNA during reverse transcription (99). The report also showed that EVs from HIV-infected cells, when cocultured with the primary macrophages, induced an NF-κB response (15). One study indicated that EVs from uninfected cells carry active c-Src, which, upon uptake by HIV-1-infected cells, activates EGFR and the PI3K/AKT/mTOR pathway. This signaling recruits the nuclear coactivator SRC-1 and histone acetyltransferase p300, remodeling chromatin at the HIV-1 LTR. The enhanced accessibility promotes viral transcription and production of proteins such as Gag and p24, thereby reactivating latent HIV-1 (100). EVs) from HIV-1-infected individuals stimulate macrophages to secrete galectin-1 (Gal-1). Elevated plasma levels of Gal-1 correlate with increased HIV-1 transcriptional activity and inflammatory markers. Gal-1 binds to latently infected resting CD4^+^ T cells and J-LAT cells in a glycan-dependent manner, activating the NF-κB pathway and promoting HIV-1 latency reversal. This EV-driven Gal-1 secretion links chronic inflammation with HIV-1 persistence in cART-treated individuals, highlighting its role in modulating reservoir dynamics and offering potential therapeutic targets (101).

Therefore, EVs play a complex role in HIV infection, depending on various factors, including the host immune system and viral strain. Hence, further studies are required to understand the complexity of dynamic crosstalk between HIV and EVs.

HIV-1 shows remarkable genetic diversity worldwide, resulting in multiple clades that differ in sequence, transmissibility, and disease progression. Among these, clade B predominates in North America and Europe, whereas clade C is more widespread in sub-Saharan Africa and Asia. Although no comparative studies have explored EVs) derived from different HIV-1 clades, such variations could influence EV composition and function. Since EVs mirror the molecular and physiological state of their producer cells, differences in viral–host interactions across clades may affect the nature of the RNA, protein, or signaling molecules packaged within them. These clade-specific differences could, in turn, modulate how EVs participate in immune activation or latency reactivation (102). Future comparative studies are therefore warranted to delineate the potential functional disparities between clade B- and clade C-derived EVs in the context of HIV persistence and pathogenesis.

### Role of EVs in SARS-CoV-2 infection

SARS-CoV-2 belongs to the betacoronaviridae family, having positive single-stranded RNA. This enveloped virus has the largest genome of approximately 27–32 kb. The virus can enter the host cell via the endocytic pathway and the involvement of the non-endosomal pathway. TMPRSS2 performs a crucial role in the cleavage of the S protein of coronavirus, which is essential for its entry (103, 104). In the absence of TMPRSS2, cathepsin L performs the proteolytic cleavage function. After the proteolytic cleavage, tetraspanin CD9 interacts with the viral receptor and facilitates its entry. ACE2 is the primary receptor needed for its entry into the target cell. ACE2 and CD9 are mostly abundant in kidney, lung epithelial cells, the intestine, and the bladder. Reports suggested that ACE2 and CD9 can be exported into the exosomes from infected cells, which can bind with the recipient’s cells and make them more susceptible to viral entry (105, 106). A study on exosomes from critical COVID patients also showed the development of the inflammasome in endothelial cells and increased production of IL-1β after coculturing EVs with EC, which is also associated with high viral load in patients’ blood (107). Although it is known that the N protein of coronavirus is responsible for inflammasome activation, in the literature, no reports show the presence of protein N in patients’ EVs. A recent study reported the immunomodulatory role of exosomes by enhancing the production of several cytokines, which are associated with the disease severity, after culturing them with CD4^+^, CD8^+^, and monocytes. Furthermore, this investigation unveiled the existence of SARS-CoV RNA within it (108).

The mass spectrometry analysis of EVs of mild and severe COVID-19 patients in various studies showed different protein patterns with respect to healthy donors. EVs derived from mild patients showed proteins related to activated immune response, migration of immune cells, and effector activity. On the contrary, severe patients derived EVs showed proteins related to metabolism, stress response, coagulation, complement cascade, endothelial cell dysfunction, thrombus formation, and inflammation (18). Reports also showed mild patients’ exosomes bear more CD4^+^ and MHC-II receptors than severe patients’ derived EVs (109). Therefore, EVs differ from disease stage in terms of their size, their receptors, and their cargo. One study also reported the presence of fibrinogen and tenascin in patient-derived exosomes, which, after coculturing with hepatocytic cell line Huh-7, stimulate the secretion of proinflammatory cytokines like TNF-α, CCL5, and IL-6 via the NF-κB pathway (110). This suggests the possible factors responsible for cytokine storms, which are the hallmarks of severe COVID-19. Interestingly, in COVID-19 patients, ACE^+^ EVs were detected, demonstrating the ability to impede viral entry by obstructing the interaction between the S protein and its cellular receptor (111).

One study showed that lung spheroid cell-derived exosomes (LSC-Exo) naturally enriched with ACE2 receptors can function as biological decoys to neutralize SARS-CoV-2. Upon inhalation, these ACE2-bearing exosomes effectively reached the respiratory tract, binding viral spike proteins and preventing viral entry into host cells. In preclinical models, such treatment significantly reduced viral load, lung injury, and inflammation, indicating robust prophylactic protection. Moreover, these exosomes showed broad neutralizing activity against multiple SARS-CoV-2 variants**,** suggesting potential for variant-independent protection. Overall, the findings highlight ACE2-expressing exosomes as a promising inhalable nanotherapeutic platform for frontline defense against coronavirus infections (112).

### Role of EVs in dengue infection

Dengue virus (DV) is a positive single-stranded RNA virus, enclosed within a capsid. It contains structural proteins (protein E and PRM E) and seven non-structural proteins. All these proteins perform different functions. NS5 proteins remain the most conserved protein in DENV. It is an arbovirus, dependent upon vector for its spread, mainly *Aedes aegypti* and *Aedes albopictus*. The main target of DENV is immune cells like dendritic cells, monocytes, and mast cells. Dendritic cell is the primary site for DENV replication. DENV-infected DC-derived EVs carry mRNA of ISGs and are directly associated with viral particles, which helps the virus to escape from immunosurveillance (113). One report also suggested that DENV-activated platelets have increased EV release. These EVs activate neutrophils and macrophages via CLEC5A and TLR2 pathways and induce NET formation. NET formation in dengue disease is associated with dengue hemorrhagic fever and directly impacts endothelial permeability and plasma leakage. These EVs also increase proinflammatory cytokine release through NLRP3 inflammasome and IL-1β release (114). The DV can change the composition of EVs’ content. The exosome population was found to be higher in DENV-infected C6/36 cells compared to mock-infected cells. Also, this study showed viral-like particles in the EVs, the transfer of which induced infection in naïve cells (115). Vora et al. reported the presence of a full-length genome that can be transported to mammalian cells. They also showed increased EVs, supporting the notion that viral RNA or its proteins are secure in a compact structure, enabling them to fuse with the recipient cells (116). Wu et al. demonstrated the presence of autophagy marker LC3 II and some viral proteins like protein E, prM/M in vesicles released from DENV-infected Huh7 cell line, which can help them to escape from host neutralizing antibodies and the dissemination of viral spread (117). EVs play crucial roles in influencing the cellular function in different pathological processes. However, there is less clarity on how plasma EVs influence immune cells’ proliferation, activation, and functions during DV infection. Our recent studies addressed this issue and reported that EV cargo may vary depending on the disease condition. We also noted that EVs released from the plasma of mild and severe dengue patients differ in their source cells; severe plasma mostly harbors EVs of platelets, whereas from mild plasma, CD3^+^ EVs were predominant. The EVs from severe dengue patients’ plasma (SD-EV) inhibited PBMC proliferation, perturbed the CD4:CD8 ratio in PBMC, and exerted apoptosis on CD4^+^ T cells. These SD-EVs showed suppression in CD4^+^ proliferation and lined the CD4^+^ subset toward IFN-γ-producing cells. We also reported that the suppression in CD4^+^ proliferation occurs through the PD-1/PD-L1 mechanism, which argues for EV-mediated silencing of the host immune response and triggering severity. We also showed different cytokines present in EVs from mild and severe dengue patients (118). EVs have emerged as critical mediators in the intercellular communication network. These vesicles encapsulate or remain associated with diverse cytokines, which play pivotal roles in modulating immune responses. The encapsulation of cytokines within EVs allows for targeted delivery to recipient cells, thereby influencing various signaling pathways that can impact viral replication and immune activation (119). Also, SD-EVs showed their immunomodulatory role on endothelial cells by affecting their migration capability. When interacting with CD4^+^ T cells, severe plasma-derived EVs induce CD44 expression and the secretion of excessive TNF-alpha, compromising endothelial cell barrier permeability (120). DENV infection in dendritic cells induces early autophagy and blocks its degradative stages, accumulating LC3^+^ vesicles. A subset of secreted EVs co-expressing LC3 carries viral RNA and facilitates cell-free transmission. Genetic variants enhancing autophagosome biogenesis increase susceptibility, highlighting the role of autophagy-derived EVs in dengue spread (121).

Recent studies highlighted a novel subset of CD4 T cells (CXCR5−PD-1^+^) detectable in severe dengue patients, which can help memory B cells differentiate into antibody-secreting cells (122). This process may trigger antibody-mediated enhancement, leading to severe dengue. Our study confirms that, apart from direct virus infection of CD4^+^ T cells, circulating EVs can modulate the expression of immunomodulatory proteins on CD4^+^ T cells.

On the other hand, several reports state the antiviral property of EVs. DENV-infected macrophage-derived EVs also included apoptotic bodies besides EVs. Different miRNAs were also expressed, besides the presence of NS3 in the EVs. Exposure of these EVs to the EA.hy926 (endothelial cells) showed the induction in TEER and increased levels of TNF-α, IP-10, IL-10, RANTES, and MCP-1 secretion. Culturing with these EVs also showed the changes in the expression of cadherin proteins in EA.hy926. This function performed by EVs highlights its antiviral response elicitation in this cell line (123). Exosomes released from DENV cellular models can deliver IFITM3 securely in the recipient cells, inhibiting viral entry into the recipient cells (124). A recent study by Vedpathak et al. aimed to examine the role of platelet-derived exosomes (PLT-EXOs) in vascular dysfunction and their association with disease severity in dengue patients (125). Using the patient’s PLT-EXOs, they demonstrated that PLT-EXOs promote vascular leakage via the release of proinflammatory mediators and compromise vascular barrier integrity in dengue patients.

## EVs AS A PROMISING DIAGNOSTIC MARKER

As EVs are a secure carrier of cargo from the origin cell. The cargo of EVs reflects the physiological status of the parental cell. The content of EVs varies at different stages of disease progression. EVs are lipid-covered entities, which gives the advantage of being used to deliver immunomodulatory cargo with suitable drugs. These present a noble intervention to treat many diseases like cancer, kidney infection, neurodegenerative disease, and several viral infections. MicroRNAs (miRNAs) are 18–25 nt in length and non-coding RNAs that can target mRNAs and regulate their expression. They are essential gene expression modulators by gene silencing and mediating post-transcriptional changes. Viruses are successfully able to encode different small non-coding RNAs and miRNA, which can act pro- and antivirally (HIV and HCV [126, 127]). These EVs can be used as a prognostic or vaccine model in different disease contexts, as described in Table 2. High-throughput sequencing of dengue patients, dengue with warning signs, and severe dengue patients showed that six miRNAs were differentially expressed in these patients. Increased levels of miRNA-486-5p and miR-320a in severe dengue patients are mainly associated with erythropoiesis and neutrophil activation, respectively. miR-122-5p showed increased expression in severe dengue patients, which is associated with hepatic dysfunction. But it was also observed in dengue warning sign patients, but not in other febrile illness patients, in PBMC (128). Tambyah et al. reported that miR-512-5p is highly expressed in acute dengue patients, which is associated with autophagy induction, T cell activation, and antiviral responses (129). One study observed increased levels of microRNA-150 in severe dengue disease, which is already established to play a role in hematopoiesis (130). CSF-derived exosomal miRNAs could significantly discriminate between acute encephalitis symptoms (AES) caused by different etiologies. Goswami et al. reported increased expression of miR-21-5p and miR-150-5p, which were significantly upregulated in JEV+ AES cases and can discriminate between JEV+ AES versus non-JEV AES (131). Interestingly, in the SIV neurological disease model, EVs harboring miR-21-5p trigger neurotoxicity within the brain. The EVs encapsulating miR-21-5p promote neurotoxicity in the brain of the SIV disease model (132). The stability of these miRNAs in body fluid is extraordinary, as this miRNA is encapsulated in EVs; therefore, it circulates freely and safely from any degradation activity. Thus, it can be used as a biomarker to predict the stage of viral infection.

**TABLE 2:** Clinical and translational applications of extracellular vesicles (EVs) as therapeutic and prognostic tools across diverse diseases

Purpose of EVs	Mechanism followed by EVs	Example
Diagnostic/prognostic biomarkers		
HIV	EV-encapsulated miRNAs remain stable in circulation and can serve as biomarkers for disease stage prediction and monitoring	HIV**:** upregulation of miR-1241a, miR-29a, miR-27a, miR-19b, miR-151-3p, miR-28-5p, miR-766 in primary CD4^+^ T cells (133). miR-28, miR-125b, miR-223, miR-382 associated with HIV latency (134). miR-17-5p, miR-29a, miR-20a, miR-196b, miR-1290 upregulated in HIV infection (135). Differential miR-31, miR-200c, miR-526a, miR-99a, miR-503 in rapid vs. slow progressors (136). Inverse correlation of miR-29a with viral load (137).
In cancer	Encapsulated in EVs	miR-1246, miR-21, miR-9, miR-338-3p, miR-340-5p, miR-124-3p, miR-29b-3p, miR-20b-5p (138, 139).
EVs as medicine		
Alzheimer allogenic human adipose MSC-derived exosomes (ahaMSC-Exos)	Neuroprotection, anti-inflammatory effects, and promotion of neuronal repair	NCT04388982: to assess safety and therapeutic efficacy of MSC-Exos in individuals with mild to moderate AD (140).
Cardiovascular disease (EV-enriched secretome from cardiovascular progenitor cells)	Regeneration of damaged myocardium, improvement of LV function, and reduction of inflammation	NCT05774509: evaluating safety and efficacy of three intravenous doses in patients with severe drug-refractory LV dysfunction (141).
In SARS COVID-19 human umbilical mesenchymal stem cells derived EVs	–^*a*^	ChiCTR2000030484: a potential therapeutic approach to treat lung damage in COVID patients (142).

^*a*^ “–,” no available data.

## EVs: EMPLOYING AS TRANSLATIONAL MEDICINE IN VIRAL DISEASE

EVs exhibit inherent characteristics like cargo transfer and intercellular communication, which render them attractive as vaccine candidates, holding significant potential for revolutionizing immunization approaches. As a subtype of EVs, exosomes serve as crucial mediators in cell-to-cell communication by transporting a diverse range of biomolecules, encompassing proteins and nucleic acids. They have emerged as a valuable resource in the realm of translational medicine.

Several studies aim to assess the viability of EV-based vaccines as a strategy against HIV. Pioneering this research field, Dr. Jim Xiang’s research team formulated a vaccine called Gp120-Texo by using adenoviral transfection in dendritic cells and deriving EVs from it. This vaccine induced robust and durable HIV-specific CD8^+^ T cell responses (143, 144). When this exosome vaccine was given to chronic HIV animals in models, it induced gag protein-specific cytotoxic T cells (145). A similar approach has been employed for various viruses, such as Ebola, influenza, HBV, and HCV, by using the viral Nef protein (146, 147). One recent study reported the presence of NS1 in the EVs derived from human A549 cells infected with Zika and DV, indicating their potential role in advancing vaccine development efforts (148). A recent study demonstrated that EVs released from Zika-infected cells carried envelope proteins that can adsorb envelope-specific antibodies *in vitro* and in a mouse model, thus reducing antibody-mediated enhancement (149). Mesenchymal stem cell-derived EVs gained attention due to their properties, such as wound healing, improving lung and kidney-associated diseases, and immune-modulatory properties (150–153). One recent study designed and tested the inhalable virus-like particle COVID-19 vaccine, which was stable for three months at room temperature. It was prepared by combining the SARS-CoV-2 receptor-binding domain with lung-derived exosomes. After administration of this vaccine to the mouse models, it stimulated the production of IgG antibodies specific to the RBD, elicited mucosal IgA responses, and led to the expression of Th1-like cytokines in CD4^+^ and CD8^+^ T cells within the lung (154).

Barnawal et al. reported that spike-activated dendritic cell-derived EVs successfully induced anti-spike IgG titers in mouse blood compared to dendritic cells or free spike protein treatment. After giving a booster dose within 15 days, antisera treatment reduced viral infection by 55–60% in vitro studies. These DEVs also induced CD19^+^CD38^+^ T cells in the spleen, lymph node, and CD8^+^ T cells in the bone marrow (BM) and spleen (155). In a recent study of exosomes, they were harnessed as a delivery system for a bivalent protein-based vaccine, incorporating two distinct viral proteins. Through genetic engineering, EVs were modified to express the SARS-CoV-2 delta spike protein or the most conserved nucleocapsid protein on their outer surface. Administration of the individual EV vaccines or their combination resulted in robust immunization, eliciting potent humoral and cellular immune responses. These findings suggest that the stealthX EVs platform holds significant promise for transforming vaccinology by leveraging the benefits of mRNA and recombinant protein vaccines, leading to the development of an advanced, swiftly producible, and low-dosage vaccine that can induce potent and broader immune responses (156).

Besides developing a vaccine, bone marrow-derived EVs (BM-EVs)) have been tested for antiviral effects against JEV in a mouse model. Treatment of JEV-infected mice with BM-EVs significantly delays survival, but cannot protect the blood-brain barrier (157).

EVs display a broad and overlapping range of physicochemical properties—such as size, composition, membrane structure, cargo, and surface interactions—and this heterogeneity poses significant challenges for their definition, isolation, manufacturing, and clinical translation. Scientists in the EV field strongly believe that advancing EV-based applications will require rigorous characterization of product quality attributes, standardization of bioprocessing methods, and new technologies that can resolve EV subpopulations with reproducible functionality (158).

As research advances, EV-based vaccines may become a valuable addition to the arsenal of preventive and therapeutic strategies against infectious diseases. However, further studies and clinical trials are necessary to understand their safety, efficacy, and scalability fully. With ongoing research and development, they hold exciting prospects for revolutionizing the field of vaccinology and improving global healthcare.

The therapeutic value of EVs relies on their content, encompassing proteins, RNAs, and lipids. However, loading specific cargo into EVs efficiently and precisely is difficult. Modifying EVs to carry desired cargo presents challenges like low loading efficiency, potential changes in EV formation, and unintended cargo alterations. Ensuring consistent and relevant cargo loading remains a significant hurdle. Due to source cell disparities, culture conditions, and isolation protocols, EV isolation techniques like ultracentrifugation and precipitation can result in variable purity, size distribution, and cargo composition. This inconsistency can impact therapeutic effectiveness due to variable EV populations with differing functions. In conclusion, while EVs hold immense promise as therapeutic agents, their clinical translation is hindered by limitations such as batch-to-batch variability, cargo loading challenges, and off-target effects. Overcoming these hurdles requires robust scientific research, innovative technological solutions, and a comprehensive regulatory framework.

## CONCLUSION

EVs, membrane-enclosed entities, possess a crucial advantage in protecting and transporting their cargo to recipient cells, facilitating biological functions such as cellular communication and immune modulation. These EVs can differ based on the specific tissue, cell type, and organ they originate from, adapting to the physiological environment they encounter. Interestingly, viruses have evolved to exploit this process for their transmission within the host. They cleverly hijack their cargo-loading machinery, incorporating their RNA, miRNA, and proteins, utilizing EVs to spread their infection effectively.

The dual role of EVs in viral infection is the focus of this review. On one hand, they can act as viral messengers, promoting the pathogenicity of the virus. On the other hand, they contain antiviral molecules capable of inducing an immune response in uninfected cells, displaying immunomodulatory properties. These features make them intriguing candidates for cell-free therapeutics approaches to combat viral diseases, as demonstrated in studies exploring EV-based vaccines for infectious diseases. However, there is still a long way to go before EVs can come as cell-free therapeutics in viral infection, as more rigorous control on EV production and cargo composition needs to be standardized. However, EVs help understand disease pathogenesis and pave the way to look into the disease.
